# Key success factors for the implementation of quality management systems in developing countries

**DOI:** 10.4102/ajlm.v12i1.2058

**Published:** 2023-01-31

**Authors:** Iryna Tanasiichuk, Olha Karaman, Larysa Natrus

**Affiliations:** 1 Department of Modern Technologies of Medical Diagnostics and Treatment, Institute of Postgraduate Education, Bogomolets National Medical University, Kyiv, Ukraine; 2 Laboratory of Oncoimmunology and Design of Tumor Vaccines, R.E. Kavetsky Institute of Experimental Pathology, Oncology and Radiobiology, National Academy of Sciences of Ukraine, Kyiv, Ukraine

**Keywords:** laboratory quality, accreditation of medical laboratories, ISO 15189, strengthening laboratory quality management systems, quality improvement

## Abstract

**Background:**

Despite the tremendous progress made in advancing laboratory medicine in low- and middle-income countries (LMICs), inadequate quality management systems (QMSs) remain a problem and barrier to provision of reliable laboratory services in resource-limited settings. Therefore, it is useful to study the experience of medical laboratories in LMICs that have successfully implemented QMS.

**Aim:**

This review identified key success factors (KSFs) for medical laboratories in LMICs implementing QMS in accordance with the International Organization for Standardization standard 15189 as a pathway to improving laboratory quality.

**Methods:**

Applying Preferred Reporting Items for Systematic Reviews procedures, we conducted a targeted search of studies from LMICs published between 2012 and 2022 to identify KSFs. Thirty-two out of 952 references retrieved were considered relevant and included in this review. Grounded theory was used to extract key features of the included studies to derive KSFs.

**Results:**

Ten KSFs for medical laboratories striving to implement QMS were identified and described. These KSFs were integrated to create a model of success for laboratory QMS implementation. The model consists of three underlying factors, namely preparing for change, resource availability, and effective project management, each comprising three separate KSFs. Institutional commitment was identified as the core of the model and is integral to ensuring the quality of laboratory services.

**Conclusion:**

Laboratories planning to implement a QMS can benefit from understanding the KSFs demonstrated in this study as this would help them to identify the necessary changes to implement and set realistic expectations about the outcomes of QMS implementation.

## Introduction

Healthcare systems around the world are tasked with improving the quality of medical care and patient safety. High-quality laboratory services are critical to achieving this goal, considering the significance of laboratory testing results in physicians’ decision-making processes.^1,2,3^

Currently, the International Organization for Standardization (ISO) 15189:2012 ‘Medical laboratories – Requirements for quality and competence’ standard^4^ (hereinafter referred to as ‘the standard’) is the most demanding regulatory document related to medical laboratories. Following the standard requires meeting both the principles of ISO 9001:2008^5^ for implementing a quality management system (QMS) and the requirements of ISO/International Electrotechnical Commission 17025:2005^6^ for technical competency. Numerous medical laboratories that have met the standard’s requirements and received accreditation have shown improvements in the quality of medical services and patient safety.^1,7,8,9,10,11,12,13^

At the same time, it is noteworthy that implementation of the standard is not an easy task, and preparation for accreditation is typically considered a multi-year, expensive, and labour-intensive project, even in developed countries.^7,8,9,12,14^ Establishing and maintaining the standard’s requirements presents significant and often insurmountable challenges in resource-poor settings.^3,7,15,16,17^

Starting with the Maputo declaration in 2008, global efforts to strengthen laboratory medicine in low- and middle-income countries (LMICs) and the unprecedented increase in international funding for these initiatives has made it possible to significantly improve the quality of laboratory services in LMICs.^18,19^ However, despite these tremendous advances in laboratory medicine, inadequate QMSs remain a problem and one of the barriers to the provision of reliable laboratory services in many LMICs.^3,15,16^ Therefore, it is useful to study the experience of medical laboratories in LMICs that have successfully implemented QMS and improved laboratory quality.

Awareness of the factors that affect the success of a QMS implementation would help identify the main steps that medical laboratories or their parent organisations (hereinafter referred to as ‘the implementers’) need to take in implementing necessary changes. These factors are actively discussed in the literature. However, the respective studies are predominantly focused on individual laboratories, or a small number of laboratories located in specific countries. A systematic review would provide a balanced and unbiased summary of the accumulated studies.

Consequently, this research aimed to identify the key success factors (KSFs) for medical laboratories in LMICs striving to implement QMS in accordance with ISO 15189 as a pathway to improving laboratory quality.

## Methods

### Reporting guidelines

The Preferred Reporting Items for Systematic Reviews (PRISMA) was used to guide the reporting of this review.^20^ This systematic review is registered in PROSPERO with the registration number: CRD42022338151.

### Literature search

For this systematic review, we searched Medline (i.e., PubMed), Web of Science, and Google Scholar in June 2022. The terms ‘ISO 15189 accreditation’, ‘laboratory quality management system’, and ‘medical laboratories’ were used in various combinations with the following words: ‘success’, ‘strengthening’, ‘improvement’, and ‘implementation’. Additional search terms, namely ‘Africa’, ‘Asia’, ‘low-income countries’, and ‘countries with limited resources’ were used to narrow down the search results. The results were cross-referenced with the World Bank’s 2022 list of low-, lower-middle-, and upper-middle-income countries.^21^ The reference lists of all the selected studies were thoroughly inspected to identify additional studies of interest.

### Study selection and data extraction

Two reviewers screened the titles and abstracts against the eligibility criteria. All the potentially relevant articles were accessed in full-text format. Both reviewers independently made the final decision on whether to include each of the articles in this review, and conflicting decisions were discussed and resolved. The articles were subjected to content analysis by two reviewers, each of whom extracted and documented the key findings of the included studies.

### Inclusion сriteria

Articles that were published in English between 2012 and 2022 and contained primary data that demonstrated the results of QMS implementation in medical laboratories in LMICs according to the standard’s requirements were included in the review. No restrictions were placed on the research design.

### Exclusion сriteria

We excluded studies without a direct focus on the implementation of the standard’s requirements, as well as studies whose reports of such implementation did not include information about the immediate evidence of improvement in the quality of laboratory services. We also excluded studies conducted in non-medical laboratories (forensic, research, etc.).

### Data synthesis

We used the grounded theory to identify the KSFs, with the data analysed using a multistage procedure of open, axial and selective coding.^22^ Conceptualisation and data categorisation were done by one of the reviewers, after which another reviewer assessed the theoretical relevance of the selected categories. Any discrepancies in the reviewers’ definition, formulation or integration of the categories were resolved through discussions until an agreement was reached.

### Assessment of the study quality

One reviewer critically evaluated the methodological quality of the papers using the critical appraisal tool described by Hawker et al.^23^ According to the tool, studies can be assessed based on nine criteria: abstract and title; introduction and aims; method and data; sampling; data analysis; ethics and bias; results; transferability or generalisability; implications and usefulness. Each criterion was evaluated on a scale of 1 (very poor) to 4 (good) in accordance with the developed protocol.^23^ Thus, each article could receive from 9 to 36 points, indicating the methodological rigour of the study. Another reviewer cross-checked 30% of the assessed studies and expressed full agreement with the initial assessment of their quality.

## Results

### Literature search results

**FIGURE 1:**
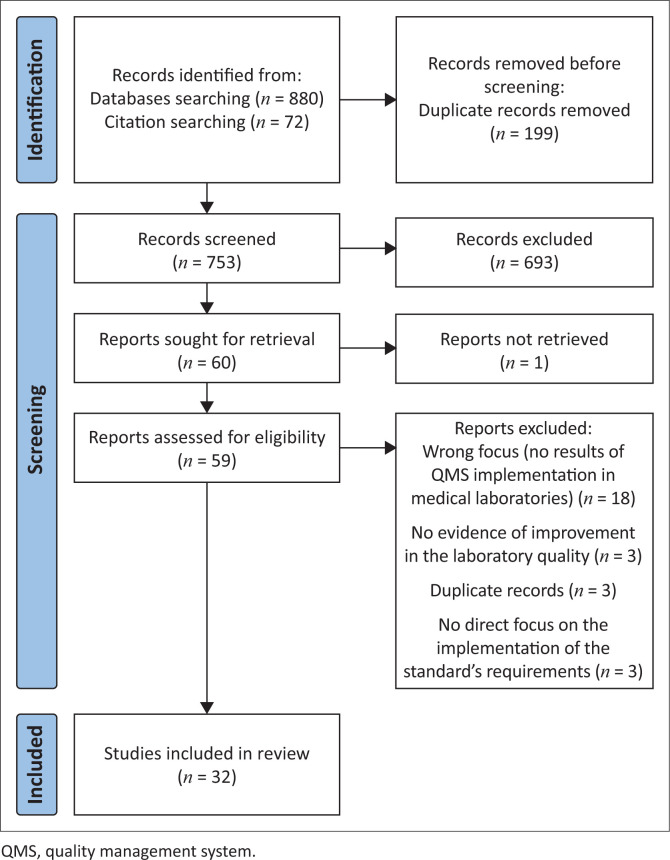
Flow diagram illustrating the literature review process to identify the key success factors for medical laboratories in low- and middle-income countries striving to implement a quality management system in accordance with International Organization for Standardization standard 15189, 2012–2022.

A total of 952 records were identified through databases (*n* = 880) and citation (*n* = 72) searching. After removing the duplicates, 753 abstracts were retrieved and screened. After applying the inclusion and exclusion criteria, two independent reviewers narrowed down the references to 59 full-text versions of articles. The analysis of those articles led to 32 of them being included in the final analysis (Figure 1).

### Characteristics of the identified studies

Most of the studies included in this review were conducted in Africa: Ethiopia (*n* = 6), Kenya (*n* = 4), Tanzania (*n* = 3), Lesotho (*n* = 3), Botswana (*n* = 2), Nigeria (*n* = 2), Cameroon (*n* = 2), Mozambique (*n* = 2), Rwanda (*n* = 1), Zambia (*n* = 1), Ghana (*n* = 1), Zimbabwe (*n* = 1), and Benin (*n* = 1).^24,25,26,27,28,29,30,31,32,33,34,35,36,37,38,39,40,41,42,43,44,45,46,47,48,49^ Studies reporting data from the Southeastern Asian countries (Cambodia [*n* = 2], Vietnam [*n* = 2]),^50,51,52,53^ the Caribbean Region (*n* = 1),^54^ and Armenia (*n* = 1)^55^ were also included in the analysis (Supplementary Table 1). We found no relevant studies conducted in Eastern Europe (Ukraine, Belarus, Moldova, Romania, etc.) or Central Asia (Kazakhstan, Uzbekistan, Kyrgyzstan, Tajikistan, Turkmenistan).

The 32 studies included in this review demonstrated the results of successful QMS implementation in 280 medical laboratories; 266 showed quality improvement and 14 met the standard’s requirements and achieved accreditation (Table 1). Two hundred and seventy-eight out of the 280 laboratories were in the public sector. Most (*n* = 237) of the studies were conducted in laboratories enrolled in the Strengthening Laboratory Management Toward Accreditation programme.

**TABLE 1:** Characteristics of laboratories that showed quality improvements in 32 identified studies conducted in low- and middle-income countries between 2012 and 2022.

Characteristics	Laboratories (*n* = 280)		Studies (*n* = 32)	
	*n*	%	*n*	%
**Geographical region**				
Africa	240	85.7	26	81.3
Southeast Asia	34	12.1	4	12.5
Caribbean Region	5	1.8	1	3.1
Armenia	1	0.4	1	3.1
**Result of QMS implementation**				
Quality improvement (increase in baseline scores)	266	95.0	22	68.8
Achievement of accreditation to ISO 15189	14	5.0	10	31.2
**Healthcare sector**				
Public	278	99.3	30	93.8
Private	2	0.7	2	6.2
**Enrolment in the SLMTA programme**				
Yes	237	84.6	24	75.0
No	43	15.4	8	25.0

QMS, quality management system; SLMTA, Strengthening Laboratory Management Toward Accreditation; ISO, International Organization for Standardization.

### Study quality

The included studies varied in their methodological quality: two studies received the highest attainable score – 36 points, 13 scored between 33 and 35 points, 16 scored between 29 and 32 points, and just one scored 25 points. Studies lost points mostly due to insufficient presentation of the methods of research (*n* = 5; 16%) and result bias (*n* = 22; 69%).

### Model of success for laboratory quality management system implementation

The use of open and axial coding techniques^22^ allowed the identification of 10 different categories, which represent the necessary activities, conditions, and strategies needed to improve the quality of laboratory services by implementing QMS in accordance with the standard. Those categories were named ‘key success factors’ as they are crucial to the implementation of the standard’s requirements (Table 2). Across the 32 studies, the most frequently identified KSFs were ‘mentorship’ (*n* = 27; 84%) and ‘trained laboratory staff’ (*n* = 26; 81%), while the least common factors were ‘personnel management’ (*n* = 8; 25%) and ‘QMS implementation strategy’ (*n* = 8; 25%).

**TABLE 2:** Key success factors for medical laboratories striving to implement a quality management system as identified by a review of 32 studies conducted in low- and middle-income countries between 2012 and 2022.

№	Key success factor	Definition	Studies reporting key success factor		
			Reference	*N*	%
1	Mentorship	Presence of experienced individuals providing organisational and consultation assistance at the workplace during the stages of QMS implementation	25,26,27,28,30,31,32,33,34,35,36,37,39,40,42,43,44,45,46,47,48,49,50,51,52,53,54	27	84
2	Trained laboratory staff	Availability of management and technical laboratory staff with the knowledge and skills necessary for the implementation, sustainability, and improvement of QMS in accordance with the standard’s requirements	24,26,27,28,29,30,31,32,34,35,36,37,39,40,42,43,44,46,47,48,49,50,51,52,54,55	26	81
3	Institutional commitment	Dedication of governmental institutions/ healthcare regulators to the goal of laboratory service quality improvement	24,25,26,27,29,31,33,36,38,39,40,41,42,48,51,53,54	17	53
4	Hospital management dedication	Top hospital management’s awareness of the need for laboratory QMS improvement and strengthening; hospital administration support	25,28,29,31,37,39,40,42,43,44,45,49,51,52,53,54	16	50
5	Staffing	Stable number of laboratory staff necessary to effectively deal with the workload; timely detection and management of personnel turnover	25,28,29,30,31,32,35,37,40,41,42,43,49,54,55	15	47
6	Physical facilities	Availability of infrastructural, financial, and material resources to ensure the implementation, sustainability, and constant improvement of laboratory QMS	28,29,30,31,34,35,39,40,43,45,48,51,54	13	41
7	Personnel motivation	Constant stimulation of laboratory personnel to perform at high levels and diligently perform their work responsibilities	26,28,30,31,37,41,42,44,45,47,48,49,53	13	41
8	Laboratory personnel commitment	Acceptance of organisational goals and rules by laboratory personnel; readiness to achieve these goals despite obstacles and hardships	24,26,29,30,34,37,38,39,40,41,43,45,52	13	41
9	Personnel management	Effective organisation, coordination, and control of the laboratory personnel’s work; laboratory manager leadership	26,28,29,30,34,37,51,52	8	25
10	QMS implementation strategy	Presence of a precise strategic plan and roadmap for QMS implementation, which allows for gradual step-by-step quality improvement	27,29,44,45,48,51,53,54	8	25

QMS, quality management system.

**FIGURE 2:**
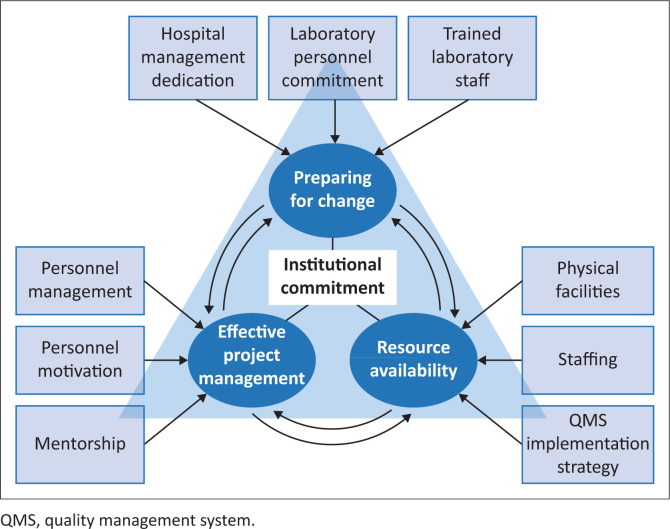
Model of success for laboratory quality management system implementation developed based on a review of 32 studies conducted in low- and middle-income countries between 2012 and 2022.

Using the selective coding technique,^22^ the 10 KSFs were integrated based on their properties and paradigmatic connections into a ‘model of success for laboratory QMS implementation’ (Figure 2). This model consists of three underlying factors, namely preparing for change, resource availability, and effective project management, each comprising three separate KSFs. These three underlying factors are united around their own central factor, which serves as a stabilising factor and the core of the model of success, without which other factors are ineffective.

### Preparing for change as the first underlying factor in the model of success for laboratory quality management system implementation

Hospital management dedication to laboratory QMS implementation, laboratory personnel commitment to quality improvement, and trained laboratory staff are the three KSFs that ensure readiness for change among the contributing parties.

A high level of readiness for change is crucial to the implementation of any organisational change,^56^ including in the medical sphere,^57,58^ and medical laboratories are no exception. The starting point of QMS implementation and laboratory quality improvement should be sufficient preparation for the implementation initiative. Sufficient preparation means that all stakeholders involved in the process of QMS implementation must agree that there is a need for change and possess the required theoretical and practical knowledge.

Firstly, the need for change must be recognised at the top management level to ensure the provision of essential financial support^25,28,40,45,52^ and organisational conditions.^29,37,40,44,51,52^ Nevertheless, top hospital management can only provide the required assistance when there is a clear understanding of the benefits of effective QMS to the patients and the facility in general.^28^ The lack of such understanding creates a strong administrative barrier to the laboratory’s quest for accreditation.^31^

At the same time, the rejection of new standards and resistance to change^29,31,39,43^ by laboratory personnel could nullify the efforts of senior management aimed at implementing laboratory QMS. Laboratory personnel commitment to quality improvement is thus one of the KSFs for the improvement of laboratory service quality.^24,26,29,30,34,37,38,39,40,41,43,45,52^ However, one must realise that the implementation of the standard’s requirements in medical laboratories often calls for considerable changes in personnel’s daily routines. Reorganising the existing processes and introducing new ones during routine laboratory practice often leads to increases in workload.^25,29,31,41,42,45,53,55^ This could significantly impact personnel’s attitude to change, more so when the personnel do not see the need for organisational changes. Involving personnel in the decision-making process and teaching them the basics of QMS could help overcome the reluctance to change and develop personnel commitment.^29,30,37,38,40,49^

Staff awareness of QMS is another component of the organisational readiness to implement changes and is also one of the two most common KSFs in the selected studies. Implementing laboratory QMS requires ensuring proper theoretical and practical preparation in advance. Managerial and technical laboratory personnel must be conversant with the standard’s requirements and practical applications.^24,27,28,29,30,31,34,37,39,40,42,43,44,46,47,48,49,50,51,52,54,55^ Technical personnel should also be aware of the standard’s requirements for technical procedures,^22,24^ and laboratory management personnel must be familiar with organisational management, leadership, and improvement activities.^26,32,50,51^

### Resource availability as the second underlying factor in the model of success for laboratory quality management system implementation

Physical facilities, staffing and QMS implementation strategy are the three main resources that could either facilitate or inhibit the overall laboratory improvement process.

Physical facilities represented by adequate infrastructure^26,29,30,31,39,48^ and independent financial^28,29,30,34,35,43,45,48,51,54^ and material^29,31,40,47,48^ resources are basic requirements for good laboratory practice. The implementers must understand that substantial investments are necessary to comply with the technical requirements of the standard regarding laboratory and office facilities, environmental conditions, and laboratory equipment, reagents, and consumables.

Adequate staffing is the second valuable resource that greatly affects the QMS implementation process. The lack of human resources^25,28,29,37,42,55^ and employee turnover^30,31,32,40,41,49,54^ can hinder the progress of QMS implementation. Laboratory management should keep this in mind and make appropriate management decisions in hiring the laboratory staff needed to efficiently handle the workload and prevent staff turnover.

The QMS implementation strategy is another essential resource that affects the efficiency of the system’s implementation. It is very important to choose the type of implementation that best fits the laboratory. Our study shows that the use of a gradual, step-by-step QMS implementation approach, such as the World Health Organization’s ‘Laboratory Quality Stepwise Implementation’ tool^51^ or the US Centers for Disease Control and Prevention and the World Health Organization – Regional Office for Africa’s ‘Stepwise Laboratory Quality Improvement Process Towards Accreditation’ framework,^29,44,45,48,54^ facilitates a laboratory’s success towards accreditation. For laboratory personnel, the unassisted procedure of creating an implementation plan is often problematic and could lead to demotivation and abandonment of the quality improvement project. The aforementioned tools provide a well-structured roadmap for QMS implementation, thus mitigating the lack of personnel experience in planning. The use of the gradual step-by-step approach of QMS implementation is also one way of dealing with the problem of financial and human resource shortage.^29,44,45,48,51,54^

### Effective project management as the third underlying factor in the model of success for laboratory quality management system implementation

Effective project management integrates the KSFs that are directly related to within-laboratory processes of QMS implementation, sustenance, and improvement, including personnel management, personnel motivation, and mentorship.

The implementation of laboratory QMS, as well as any other quality systems, requires effective human resource management and leadership, which requires laboratory leaders to have the appropriate knowledge, skills, and abilities.^26,28,29,30,34,37,51,52^ The establishment of a managerial infrastructure and delineation of management responsibilities increase the efficiency of personnel management and contribute to the success of change implementation in medical laboratories.^28,29,30,34,37^

Forty-one percent of the analysed studies revealed personnel motivation as key to successful QMS implementation.^26,28,30,31,37,41,42,44,45,47,48,49,53^ Our results correspond to the findings of other studies that have recognised the psychological aspect of organisational changes and individual change acceptance as key components of success in healthcare innovation.^57,58^

Motivating and inspiring the personnel to act towards achieving a common goal is the laboratory managers’ responsibility, and this requires strong leadership skills. The problem is that most of the laboratory leaders have not received specific training in this area.^59^ At the same time, our study shows that it is important for laboratory managers to develop their leadership skills^32,52^ and demonstrate their commitment to quality by establishing a shared vision and encouraging employees to do their best to improve the quality of laboratory services.^24,26,30^ The lack of leadership skills among laboratory managers requires appropriate measures to be taken to solve this problem. One of the possible mechanisms to overcome this shortcoming can be mentorship.

In 27 of the 32 studies included in this review, mentorship was identified as one of the KSFs for executing laboratory service quality improvement projects and receiving accreditation.^25,26,27,28,30,31,32,33,34,35,36,37,39,40,42,43,44,45,46,47,48,49,50,51,52,53,54^ The mentor’s duty is not only to ensure the QMS implementation by planning and monitoring quality improvement activities^27,32,40,50^ and increasing the staff awareness of QMS implementation mechanisms,^27,35,36,40,45,46,50,51,54^ but also to influence the attitude of laboratory managers to advocate and support all quality improvement efforts.^28,32,33,35,46,51,52,54^ Thus, the implementers may consider mentorship as one of the additional tools of laboratory QMS implementation, especially when dealing with personnel unpreparedness for change and weak leadership.

### Institutional commitment as the central factor in the model of success for laboratory quality management system implementation

Fifty-three percent of the studies included in this review convincingly show that awareness of the essential role of laboratory medicine in a functioning healthcare system at the policy and governmental levels and the appropriate facilitation of the accreditation process of the medical laboratory are crucial factors in improving the quality of laboratory services.^24,25,26,27,29,31,33,36,38,39,40,41,42,48,51,53,54^

Our study shows that commitment to laboratory quality improvement in LMICs must come from the top down. If the healthcare regulators are not truly dedicated to improving laboratory service quality, any efforts by individual laboratories to achieve ISO 15189 accreditation will fail. Nevertheless, some accreditation requirements could be achieved by the laboratories on their own. For example, all procedures required by the standard can be documented, internal audit processes can be implemented, quality indicators can be established, and the inventory control system for reagents and consumables can be introduced. All of this will undoubtedly lead to some improvements in laboratory quality. However, for full compliance with the standard’s requirements, laboratories are dependent on the government.

The medical laboratories in LMICs are faced with common problems such as shortage of robust supply chains for reagents and consumables,^26,31^ deficit of equipment maintenance providers,^29,31,40,47,48^ lack of external quality assurance,^24,40,48^ insufficient workforce capacity,^25,29,31,32,37,40,41,42,49,55^ limited number of trained laboratory personnel,^25,26,29,30,54^ etc. These difficulties make it nearly impossible to meet the standard’s requirements. It is important to realise that resolving these issues is not under the control or purview of any single laboratory management team and requires governmental intervention. If these problems are far from being resolved, expecting a laboratory to achieve an internationally recognised accreditation will often be an unreasonable initial goal.

Thus, an understanding of the policy environment would allow the implementers to have realistic expectations about the outcomes of QMS implementation. This understanding is also important to guide the choice of an implementation strategy, the development of effective timetables, and the decisions about the involvement of international or local partners.

## Discussion

This study shows that tangible laboratory quality improvement is achievable when the implementers can ensure а high level of staff readiness for change, resource availability, and effective project management. These three underlying factors in the model of success for laboratory QMS implementation are attainable even in resource-limited settings. However, for full compliance with the standard’s requirements, certain conditions must be guaranteed at the policy and governmental levels. A top-down approach, which implies an initial dedication of governmental institutions, is critical for improving laboratory service quality and achieving ISO 15189 accreditation in LMICs. Thus, institutional commitment is the central factor stabilising the model of success for laboratory QMS implementation and is integral to ensuring the quality of laboratory services.

This study had several limitations, the first of which is the small number of geographical regions included. Most of the studies included were conducted in African countries. No studies from Eastern Europe or Central Asia could be identified. Another limitation was that most of the studies included were on laboratories that had participated in the Strengthening Laboratory Management Toward Accreditation programme, thus making it difficult to assess the success factors for improving laboratory service quality independently of the Strengthening Laboratory Management Toward Accreditation programme. Another limitation was the varying level of methodological quality of the included studies. Studies that scored ‘poor’ or ‘very poor’ on any of the nine assessed criteria were not excluded from this review. Due to the large heterogeneity of the design and research outcome of the included studies, we could not conduct a meta-analysis or comparisons across studies.

Despite these limitations, this study has demonstrated that laboratories planning to implement a QMS can benefit from understanding the KSFs identified in this study as this would help to identify the main steps they need to take in implementing the necessary changes and set realistic expectations about the outcomes of QMS implementation.
